# Supplementary material for: Fibrilar Polymorphism of the Bacterial Extracellular Matrix Protein TasA
Source: Microorganisms. 2021 Mar 4;9(3):529. doi: 10.3390/microorganisms9030529 (PMC8000256; doi:10.3390/microorganisms9030529)
Supplement: Supplementary file 1 [file microorganisms-09-00529-s001.pdf]

microorganisms

MDPI

Article

Fibrilar Polymorphism of the Bacterial Extracellular Matrix Protein TasA

Mnar Ghrayeb [1], Shahar Hayet [1], Neta Lester-Zer [1], Yael Levi-Kalisman [2,3] and Liraz Chai [1,2,*]

[1] Institute of Chemistry, the Hebrew University of Jerusalem, Edmond J. Safra Campus, Jerusalem 91904, Israel; mnar.ghrayeb@mail.huji.ac.il (M.G.); shahar.hayet@mail.huji.ac.il (S.H.); neta.lester@mail.huji.ac.il (N.L.-Z.)

[2] The Center for Nanoscience and Nanotechnology, The Hebrew University of Jerusalem, Edmond J. Safra Campus, Jerusalem 91904, Israel; yael.kalisman@mail.huji.ac.il

[3] The Institute of Life Sciences, The Hebrew University of Jerusalem, Edmond J. Safra Campus, Jerusalem 91904, Israel

* Correspondence: Liraz.chai@mail.huji.ac.il; Tel.: +972-2-6585303, Fax: +972-2-5660425

Supporting information

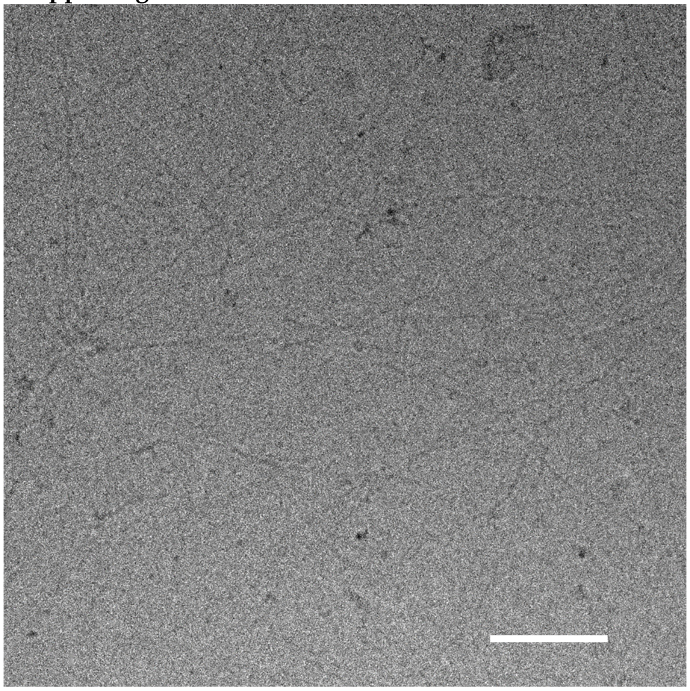

Figure S1. Cryo-TEM image of TasA fibers that formed at 700 μg/ml protein, 50 mM NaCl, using preparation method 2. Scale bar corresponds to 100 nm.

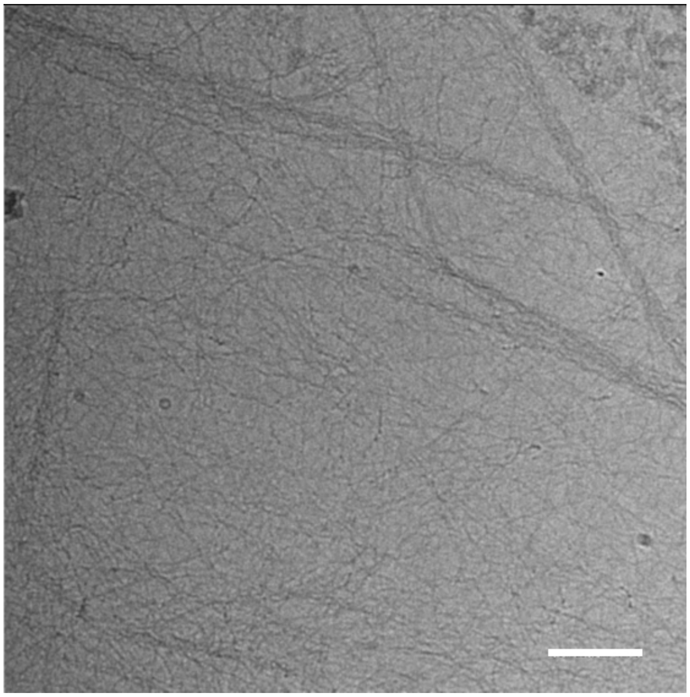

Figure S2. TasA bundles formed at 2 mg/ml protein, 2M NaCl, using preparation method 2. Scale bar corresponds to 100 nm.
